# Molecular detection of *Babesia* spp. in dogs in Germany (2007–2020) and identification of potential risk factors for infection

**DOI:** 10.1186/s13071-023-06005-7

**Published:** 2023-11-02

**Authors:** Ingo Schäfer, Christina Sabine Helm, Georg von Samson-Himmelstjerna, Jürgen Krücken, Tanja Kottmann, Annette Holtdirk, Barbara Kohn, Guy Hendrickx, Cedric Marsboom, Elisabeth Müller

**Affiliations:** 1 LABOKLIN GmbH & Co. KG, Steubenstraße 4, Bad Kissingen, 97688 Germany; 2 Institute of Parasitology and Tropical Veterinary Medicine, Freie Universität Berlin, Königsweg 67, Berlin, 14163 Germany; 3 Clinical Research Organization Dr. med. Kottmann GmbH & Co. KG, Beverstraße 64, Hamm, 59007 Germany; 4 Small Animal Clinic, School of Veterinary Medicine, Freie Universität Berlin, Oertzenweg 19b, Berlin, 14163 Germany; 5 R&D Department, AVIA GIS, Risschotlei 33, Zoersel, 2980 Belgium

**Keywords:** Canine babesiosis, Tick, *Dermacentor reticulatus*, Vector-borne disease

## Abstract

**Background:**

In Europe, canine babesiosis is most frequently caused by *Babesia canis* and *Babesia vogeli*, and occasionally by *Babesia gibsoni.*. In Germany, *B. canis* is recognized as endemic. The aims of this study were to assess how often *Babesia* spp. infections were diagnosed in a commercial laboratory in samples from dogs from Germany, and to evaluate potential risk factors for infection.

**Methods:**

The database of the LABOKLIN laboratory was screened for *Babesia* spp.-positive polymerase chain reaction (PCR) tests for dogs for the period January 2007–December 2020. Sequencing was performed for positive tests from 2018 and 2019. Binary logistic regression analysis was performed to determine the effects of sex, season, and year of testing. Questionnaires were sent to the submitting veterinarians to obtain information on travel abroad, tick infestation, and ectoparasite prophylaxis of the respective dogs. Fisher’s exact test was used to calculate statistical significance and *P* < 0.05 was considered statistically significant.

**Results:**

In total, 659 out of 20,914 dogs (3.2%) tested positive for *Babesia* spp. by PCR. Of 172 sequenced samples, *B. canis* was identified in 156, *B. vogeli* in nine, *B. gibsoni* in five, and *B. vulpes* in two. Season had a statistically significant impact on test results when summer/winter (1.6% tested positive) was compared to spring/autumn (4.7%), with peaks in April (5.2%) and October (7.4%) [*P* < 0.001, odds ratio (OR) = 3.16]. Sex (male 3.5%, female 2.8%; *P* = 0.012, OR = 1.49) and age (< 7 years old 4.0%, ≥ 7 years old 2.3%; *P* < 0.001, OR = 1.76) of the tested dogs also had a statistically significant effect. A statistically significant impact was demonstrated for observed tick attachment (*P* < 0.001, OR = 7.62) and lack of ectoparasite prophylaxis (*P* = 0.001, OR = 3.03). The frequency of positive *Babesia* spp. tests did not significantly differ between the 659 dogs that had never left Germany and the 1506 dogs with known stays abroad (*P* = 0.088).

**Conclusions:**

The possibility of canine infection with *B. canis* needs to be especially taken into consideration in spring and autumn in Germany as the activity of the tick *Dermacentor reticulatus*, a potential vector for canine babesiosis, is highest in these seasons. Travel and importation of dogs are considered major factors associated with canine babesiosis in Germany. However, autochthonous *Babesia* spp. infections also occur in a considerable number of dogs in Germany.

**Graphical Abstract:**

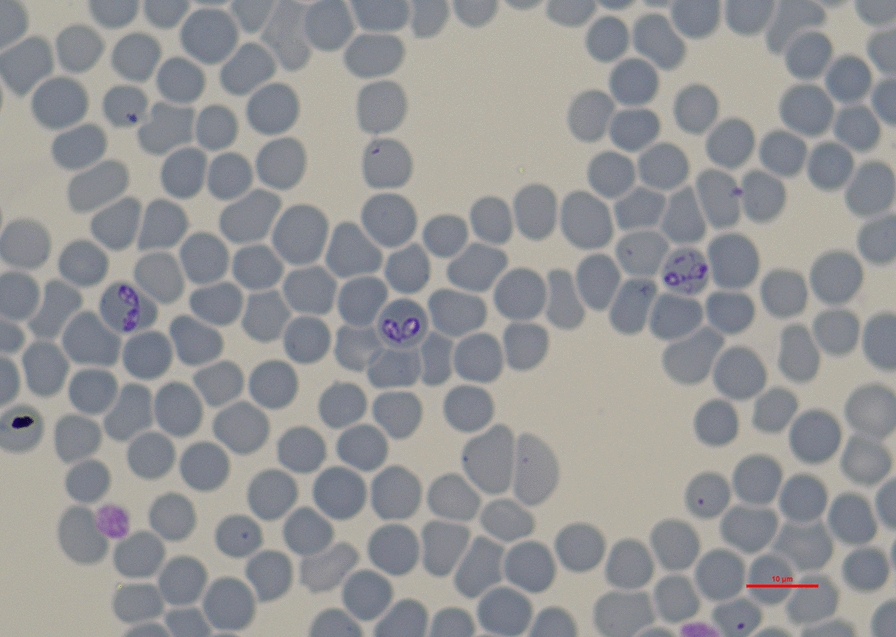

**Supplementary Information:**

The online version contains supplementary material available at 10.1186/s13071-023-06005-7.

## Background

*Babesia* spp. are vector-borne infectious hemoprotozoans that are transmitted by hard ticks and exclusively infect erythrocytes [1]. They are generally quite host specific with respect to the transmitting tick species and mammalian host, e.g. dogs. Four species of *Babesia* spp. infect dogs in Europe: *Babesia canis*, *Babesia vogeli*, *Babesia gibsoni* and *Babesia vulpes* (synonyms *Babesia annae*, *Babesia microti*-like). The distribution of these infectious agents in Europe is highly variable and largely depends on the geographical distribution of their vectors [2]. In contrast to vectorial transmission, direct transmission via blood transfusion or contamination of cannulae, and vertical transmission, have been rarely described [2–4].

*Babesia canis* is endemic in northern Spain, Italy, Portugal, France, the Netherlands as well as eastern and central Europe, including the Baltic region, and its distribution in these areas correlates with that of the vector *Dermacentor reticulatus* [5–7]. In Germany, canine infections with *B. canis* have been historically linked to stays abroad of dogs, but cases of autochthonous infection are being increasingly reported from several areas of Germany [8–16]. *Babesia canis* has been occasionally detected in *D. reticulatus* ticks in the southern federal states of Germany, e.g. Bavaria {1/301 (0.3%) ticks testing positive; [17]}, Baden-Württemberg {2/3411 (0.06%) testing positive [18]}, and Saarland {10/397 (2.5%) testing positive [19]}, but thus far has not been detected in northern federal states, e.g. Berlin and Brandenburg [20].

*Babesia vulpes*, with *Ixodes hexagonus* and *Ixodes canisuga* as the suspected vectors, has been detected in dogs in northwestern Spain, Portugal, Croatia, Serbia, France and Sweden [21–26], in foxes in Italy and Germany [27–29], and in ticks from dogs in the UK [30], which suggests that autochthonous infection in Germany is a possibility. *Babesia vogeli* transmitted by *Rhipicephalus sanguineus* has mainly been reported in the Mediterranean area [2]. Infections with *B. gibsoni* (vector *Haemaphysalis* spp., *Dermacentor* spp.; *R. sanguineus* suspected) have only been found sporadically, and are rare in Europe, where they were mainly reported for dogs with stays outside of Europe [21, 24, 31, 32]. In Germany, two autochthonous canine infections with *B. gibsoni* have been thus far reported [33]. In conclusion, *B. canis* is present in Germany and has been identified as the causal agent of autochthonous canine infections, whereas infections with *B. gibsoni* and *B. vogeli* are most likely due to stays abroad of dogs to areas endemic for these species. Canine infections with *B. vulpes* and *B. gibsoni* are rare in Europe.

The aims of this study were to assess the annual percentages of dogs testing positive for *Babesia* spp. by polymerase chain reaction (PCR) from 2007 to 2020, and to evaluate potential risk factors, e.g. stays abroad, ectoparasite prophylaxis, and tick infestation. A secondary aim was to map canine *Babesia* spp. infections in Germany from 2007 to 2020 based on samples submitted to a German veterinary diagnostic laboratory.

## Methods

### Sample identification

The database of the LABOKLIN laboratory (Bad Kissingen, Germany) was screened retrospectively for *Babesia* spp. PCR test results in dogs for the period January 2007–December 2020. Samples of blood that had been stored with the anticoagulant ethylenediaminetetraacetic acid were sent in for analysis by veterinarians in Germany. Automated nucleic acid extraction was carried out on sample volumes of 200 µL using a commercially available kit (MagNA Pure 96 DNA and Viral NA Small Volume Kit; Roche Diagnostics, Mannheim, Germany) according to the manufacturer’s instructions. The resulting nucleic acids were eluted in a final volume of 100 µL. Dogs were included in the study if an 18S ribosomal RNA (rRNA)-PCR with gel electrophoresis for the detection of *Babesia* spp. had been performed between January 2007 and December 2020 [upstream PCR primer RIB-13, 5'-CCG AAT TCT TTG TGA ACC TTA TCA-3'; downstream PCR primer RIB-3, 5'-CGG GAT CCT TC(A,G) CTC GCC G(C,T)T ACT-3']. The PCR was applied as a qualitative assay (negative/positive) [34]. Ct values below 35 were considered positive. Each PCR run included a negative and a positive control as well as an extraction control for each sample to check for nucleic acid extraction and PCR inhibition (DNA Process Control Detection Kit; Roche Diagnostics).

Only tests from the first examination or the first positive examination of the individual dogs were included in the study, with the aim of excluding follow-up examinations and/or examinations post-treatment that might have influenced the reported percentage of dogs testing positive. Samples originated either from so-called travel profiles or from individual orders, in which the submitting veterinarians ordered a *Babesia* spp. PCR separately. Travel profiles are offered by LABOKLIN to veterinarians on a fee-for-service basis for the screening of vector-borne infectious pathogens.

For the analysis of federal states, Berlin was grouped with Brandenburg, Bremen with Lower Saxony, and Hamburg with Schleswig–Holstein. For the analysis of the monthly distribution of positive test results, months were grouped into seasons (where spring comprises March, April and May; summer June, July and August; autumn September, October and November; winter December, January and February).

### Species identification

DNA was available for dogs testing positive for* Babesia* spp. from 2018 onwards. DNA isolated from samples from 2018 and 2019 which gave a positive PCR result for *Babesia* spp. was analyzed using 18S rRNA [34] {primers RLB-F (5'-GAGGTAGTGACAAGAAATAACAATA-3') and RLB-R (5'-TCTTCGATCCCCTAACTTTC-3') [35]} followed by cloning and sequencing as reported in detail recently [15]. Sequence data were analyzed using Nucleotide Basic Local Alignment Search Tool (BLASTn) in GenBank [36].

### Statistical analyses

Questionnaires were sent by post to all veterinary practices involved in the study for information on possible stays abroad, ectoparasitic prophylaxis, and tick infestations for all of the dogs included in the study. All of the metric parameters were checked for normality by the Kolmogorov–Smirnov test. Fisher’s exact test was used for categorical parameters to calculate the statistical impact of age (split into age categories of < 7 years and ≥ 7 years), sex, season, tick attachment, and ectoparasite prophylaxis on PCR results. Binary bivariate (often also called ‘simple binary bivariate’) and multiple logistic regression analysis were performed, and odds ratios (ORs) were calculated. The statistical analysis was done with SPSS for Windows (version 28.0; SPSS, Armonk, NY) and *P* < 0.05 was considered to indicate statistical significance. The 95% confidence intervals (CI) for the proportions of dogs testing positive by PCR were calculated using the Wilson procedure, including correction for continuity. Binary logistic regression analyses (bivariate and multiple) were performed to determine the effect of sex, season, and year of testing.

For the spatial distribution modelling, a set of 28 covariates was built, including the 19 BioClim variables, a temporal Fourier analysed normalized difference vegetation index, a digital elevation model, and population density. For the mapping and modelling analysis, all covariates were aggregated to the five-digit postcode level to match the disease data. All data preparation was conducted in R 4.1.2. The spatial distribution modelling was conducted in VECMAP software version 2.5.0.21152 (Avia-GIS). A random forest model, which is a machine-learning model, was trained based on the PCR results at the five-digit postcode level. After model training, the aggregated covariates were used for predictions for all five-digit postcodes in Germany. The data provided by the prediction map of the distribution were then classified according to the following five classes: very low (0.00–0.27), low (0.27–0.46), medium (0.46–0.55), high (0.55–0.73), and very high (0.73–1.00). The output was visualized in QGIS 3.16.

## Results

### Signalment of dogs testing positive for *Babesia* spp. by PCR

**Fig. 1:**
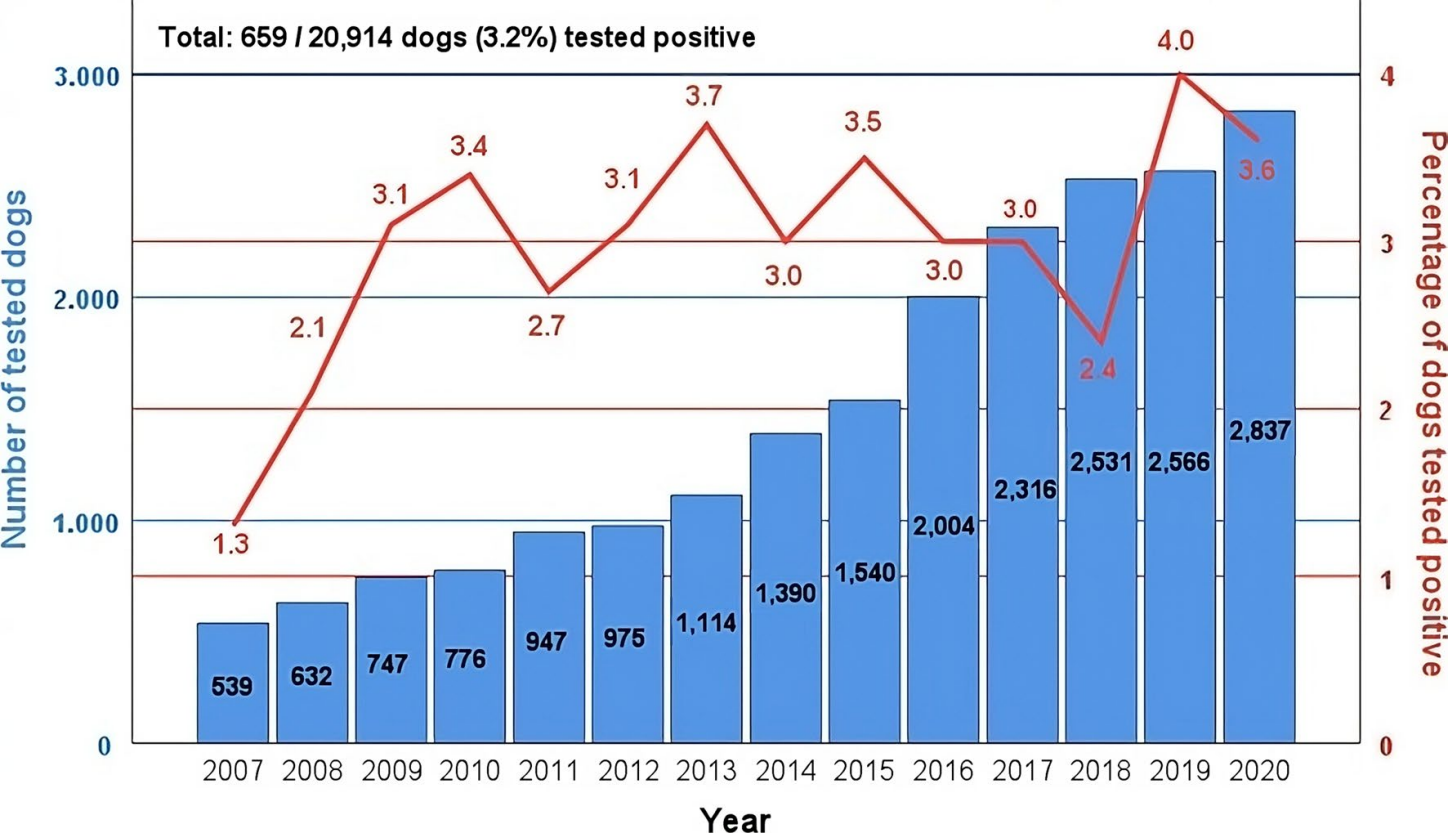
Number of dogs tested for *Babesia* spp. by polymerase chain reaction (PCR) and percentages of dogs testing positive from 2007 to 2020

In total, 659 out of 20,914 dogs [3.2% (95% CI 3.0—3.5)] tested positive for *Babesia* spp. by PCR (Fig. 1). Age was known for 18,685 of the 20,914 dogs (89.3%); the median age was 7.0 years (mean 6.6 years, range 0.2–19 years, SD 4.0 years). Of these 18,685 dogs, 580 (3.1%) tested positive for *Babesia* spp. by PCR. The median age was used to divide the study population into two age groups: < 7 years old, ≥ 7 years old. There were 9200 dogs < 7 years old, of which 363 (4.0%) tested positive. There were 9485 dogs ≥ 7 years old, of which 216 (2.3%) tested positive. The sex was known for 19,348 of the 20,914 dogs (92.5%); 9722 were female (50.2%) and 9626 were male (49.8%). In total, 277 of the 9722 female dogs (2.8%) and 335 of the 9626 male dogs (3.5%) tested positive for *Babesia* spp. by PCR.

The bivariate logistic regression analysis demonstrated a statistically significant impact of sex (male sex entered as the variable; *n* = 19,348; *P* < 0.001) and age (dogs ≥ 7 years old entered as the variable; *n* = 18,685; *P* < 0.001) on *Babesia* spp. test results. Dogs ≥ 7 years of age had lower odds [OR = 0.91 (95% CI 0.89–0.93)] than younger dogs, and males had higher odds [OR = 1.45 (95% CI 1.23–1.70)] than females for testing positive (Table 1).

**Table 1:** Bivariate logistic regression analyses in 20,914 dogs tested for *Babesia* spp. by PCR from 2007 to 2020 in the laboratory LABOKLIN (Bad Kissingen, Germany)

	***B***	***SE***	Wald	***P***	Odds Ratio	95%-CI for Odds Ratio	
						Lower bound	Upper bound
Age (n = 18,685)	– 0.093	0.011	67.258	< 0.001^***^	0.911	0.892	0.932
Sex (n = 19,348)	0.368	0.083	19.449	< 0.001^***^	1.445	1.227	1.701
Year (n = 20,914)	0.026	0.011	5.549	0.018^***^	1.027	1.004	1.049
Seasonality (spring/autumn, Year (n = 20,914))	1.107	0.091	147.894	< 0.001^***^	3.025	2.530	3.615
Prophylaxis (n = 770)	1.108	0.345	10.306	0.001^***^	3.029	1.540	5.958
Travel/import (n = 1,482)	0.302	0.173	3.035	0.081	1.352	0.963	1.898
Tick attachment (n = 868)	2.031	0.308	43.494	< 0.001^***^	7.622	4.168	13.938
Month (January)	– 0.227	0.174	1.715	0.190	0.797	0.567	1.120
Month (February)	– 0.767	0.219	12.308	< 0.001^***^	0.464	0.303	0.713
Month (March)	– 0.105	0.162	0.422	0.516	0.900	0.656	1.236
Month (April)	0.583	0.166	25.081	< 0.001^***^	1.791	1.426	2.249
Month (May)	0.327	0.119	7.576	0.006^***^	1.386	1.099	1.749
Month (June)	– 0.545	0.158	11.955	0.001^***^	0.580	0.426	0.790
Month (July)	– 1.212	0.223	29.557	< 0.001^***^	0.297	0.192	0.461
Month (August)	– 1.043	0.281	22.841	< 0.001^***^	0.352	0.230	0.540
Month (September)	0.234	0.127	3.381	0.066	1.264	0.985	1.622
Month (October)	1.027	0.102	101.758	< 0.001^***^	2.793	2.288	3.411
Month (November)	0.399	0.124	10.345	0.001^***^	1.490	1.169	1.900
Month (December)	– 0.830	0.229	13.146	< 0.001^***^	0.436	0.279	0.683

For all Wald statistics, 1 *df*

*B* Unstandardized regression weight, *CI* confidence interval

^a^Variable entered, dogs ≥ 7 years old

^b^Variable entered, male dogs

^c^Variable entered, year of testing (2007–2020)

^***^P < 0.001

### Year of testing, season, and regional distribution in Germany

Data on the years and season of testing as well as on regional distribution were available for all 20,914 dogs included in the study. In the bivariate logistic regression analysis, statistically significant impacts on *Babesia* spp. test results were demonstrated for February, April–August, and October–December. The highest odds were for October [OR = 2.79 (95% CI 2.29–3.41)], April [OR = 1.79 (95% CI 1.42–2.25)], November [OR = 1.49 (95% CI 1.17–1.90)], and May [OR = 1.39 (95% CI 1.10–1.75)]. The lowest odds were for July [OR = 0.30 (95% CI 0.19–0.46)], August [OR = 0.35 (95% CI 0.23–0.54)], December [OR = 0.44 (95% CI 0.28–0.68)], February [OR = 0.46 (95% CI 0.30–0.71)], and June [OR = 0.58 (95% CI 0.43–0.79)].

In spring/autumn, a higher percentage of dogs tested positive (494/10,570 dogs; 4.7%) compared to summer/winter (165/10,344 dogs; 1.6%). The comparison between spring/autumn and summer/winter also revealed a statistically significant impact of season on *Babesia* spp. test results (*P* < 0.001), with an OR of 3.03 (95% CI 2.53–3.62) (Table 1). The highest percentages of dogs testing positive were for 2019 (4.0%), 2013 (3.7%) and 2020 (3.6%). The lowest percentages were seen in 2007 (1.3%), 2008 (2.1%) and 2018 (2.4%) (Fig. 1). Year of testing had a statistically significant impact according to the bivariate regression [*n* = 20,914; *P* = 0.018; OR = 1.03 (95% CI 1.00–1.05)] (Table 1).

The highest percentages of dogs testing positive for *Babesia* spp. by PCR were in Saxony-Anhalt (7.7%), Saarland (7.2%) and Hesse (5.2%) (Table 3). The lowest percentages were detected in Mecklenburg-Western Pomerania (1.5%), Rhineland-Palatinate (1.7%) and Thuringia (1.7%) (Table 2). In the distribution machine learning model, moderate and high likelihoods of positive test results were predominantly for northwestern, eastern, and southern parts of Germany (Fig. 3).

**Table 2:** Distribution by German federal state for samples of 20,914 dogs tested for *Babesia* spp. by PCR from January 2007 to December 2020 that were sent in by veterinarians

Federal states	Tested dogs (*n*)	Tested positive [*n*; percentage in brackets; 95% CI lower limit, 95% CI upper limit in parentheses]
Baden-Wuerttemberg	2497	98 [4.0% (3.2, 4.8)]
Bavaria	1669	56 [3.4% (2.6, 4.4)]
Berlin/Brandenburg	2366	72 [3.1% (2.4, 3.8)]
Hesse	1836	95 [5.2% (4.2, 6.3)]
Lower Saxony/Bremen	2742	71 [2.6% (2.0, 3.3)]
Mecklenburg-Western Pomerania	412	6 [1.5% (0.6, 3.3)]
North Rhine-Westphalia	4685	109 [2.3% (1.9, 2.8)]
Rhineland Palatinate	1972	33 [1.7% (1.2, 2.4)]
Saarland	601	46 [7.7% (5.7, 10.2)
Saxony	622	21 [3.4% (2.2, 5.2)]
Saxony-Anhalt	405	31 [7.7% (5.3, 10.8)]
Schleswig–Holstein/Hamburg	768	14 [1.8% (1.0, 3.1)]
Thuringia	357	6 [1.7% (0.7, 3.8)]
Total	20,914	659 [3.2% (2.9, 3.4)]

For abbreviations, see Table 1

### Multiple logistic regression

The multiple logistic regression analysis included the PCR results for all 18,685 dogs of known age as the dichotomous dependent variable and, as the explanatory variables, the categorical variables age group (< 7 years vs. ≥ 7 years), sex, season (spring/autumn vs. summer/winter), and the metric variable year. The impact of age group was statistically significant, with dogs < 7 years old having 76% higher odds of testing PCR positive (*P* < 0.001, OR = 1.76) (Table 2). Season remained a significant variable in the multiple logistic regression analysis, with more than threefold higher odds for dogs to be *Babesia* positive in spring/autumn than in summer winter (*P* < 0.001, OR = 3.16) (Table 3). Sampling year also had a significant effect (*P* = 0.007), with each additional year having 3% higher odds than the year before (OR = 1.03) (Table 3).

**Table 3:** Multiple logistic regression analysis for the 18,685 dogs of known age tested for *Babesia* spp. by PCR from 2007 to 2020

	*B*	SEM	Wald	*P*	OR	95% CI for OR	
						Lower bound	Upper bound
Sex (male)	0.400	0.089	20.269	< 0.001***	1.491	1.253	1.775
Age (< 7 years)	0.563	0.088	41.297	< 0.001***	1.756	1.479	2.085
Years^a^	0.033	0.012	7.349	0.007**	1.033	1.009	1.058
Season (spring/autumn)	1.151	0.098	137.607	< 0.001***	3.160	2.607	3.830

For all Wald statistics, 1 *df*. For abbreviations, see Table 1

^a^Years of testing, 2007–2020

^***^P < 0.001

### Questionnaire data

Questionnaires were answered for 2165 of the 20,914 dogs (10.4%). Due to the overall lack of feedback, no multiple logistic regression model was calculated to analyse the impact of tick attachment, ectoparasite prophylaxis, and stays abroad.

Regarding tick attachment, data were available for 869 of the 2165 dogs (40.1%). Tick attachment was reported by the veterinarians for 236 of these dogs [27.2%; 39 tested positive (16.5%)]; no tick attachment was reported for the other 633 dogs [72.8%; 16 tested positive (2.5%)]. In dogs with tick attachment, an almost 8 times higher odds of testing positive by PCR was shown in the bivariate logistic regression analysis [OR = 7.63 (95% CI 4.17–13.94), *P* < 0.001] (Table 1).

Data on ectoparasite prophylaxis were available for 771 of the 2165 dogs (35.6%). Ectoparasite prophylaxis had been performed in 392 of these 771 dogs (50.9%); of these 392 dogs, 12 tested positive for *Babesia* spp. (3.1%). In the other 379 dogs, no ectoparasite prophylaxis was reported [49.1%; 33 of these 379 dogs tested positive (8.7%)]. Dogs without ectoparasite prophylaxis had 3 times higher odds of testing positive for *Babesia* spp. by PCR according to the bivariate logistic regression analysis [OR = 3.03 (1.54–5.96), *P* < 0.001] (Table 1).

Travel abroad/importation were reported for 962 of the 2165 dogs (44.4%). Of these 962 dogs, 600 had been imported [62.4%; 53 tested positive (8.8%)]; and 315 had travelled abroad [16.9%; 31 tested positive (9.8%)]; and fourty seven had been imported and also travelled abroad [2.5%; three tested positive (6.4%)]. For 905 of the 2165 dogs, no stays abroad from Germany was reported [41.8%; 62 tested positive (6.9%)]. For 298 of the 2165 dogs, it was not known if the animals had stays abroad [13.8%; 27 tested positive (9.1%)]. There was no statistically significant impact of stays abroad on *Babesia* spp. test results when data for dogs with known stays abroad [962/1867 (51.5%); 87 tested positive [9.0%)] were compared with those that had no stays abroad out of Germany [905/1867 (48.5%); 62 tested positive (6.9%)] (Fisher’s exact test, *P* = 0.088). Most of the stays abroad of the tested dogs were within the European Union, and were predominantly to Spain (*n* = 168), Romania (*n* = 135), Italy (*n* = 107), and Hungary (*n* = 80) (Additional file 1). Among those countries visited for which data on at least 50 dogs were included in the analysis, the highest frequencies of dogs testing positive were for those that had travelled to Poland (17.9%), Hungary (13.8%), and Romania (13.3%).

In dogs with known stays abroad and results of *Babesia* spp. sequencing, *B. canis* infections were only found for those that had stays abroad within central, eastern, and northern Europe, including Germany (*n* = 18), Hungary (*n* = 7), Poland (*n* = 5), France (*n* = 4), Denmark (*n* = 1), Switzerland (*n* = 1), and the Netherlands (*n* = 1). *Babesia vogeli* was exclusively detected in dogs that had stays abroad in southern European countries, namely Spain (*n* = 3) and Greece (*n* = 2). In three dogs that had travelled to Romania, *B. gibsoni* infections were identified. In the two dogs infected with *B. vulpes*, stays abroad in Spain had been reported (Table 4).

**Table 4:** Recorded stays abroad from Germany for 172 dogs for which *Babesia* spp. were successful sequenced

Country	Total	Import	Travel^a^	Import and travel^a^	*Babesia canis* (*n*), *Babesia vogeli* (*n*), *Babesia gibsoni* (*n*), *Babesia vulpes* (*n*)
No information provided	94	–	–	–	90, 3, 0, 0
No travel abroad	18	–	–	–	18, 0, 0, 0
Bosnia Herzegovina	1	–	1	–	1, 0, 0, 0
China	4	4	-	–	1, 1, 2, 0
Croatia	7	5	2	–	7, 0, 0, 0
Denmark	1	–	1	–	1, 0, 0, 0
France	4	–	4	–	4, 0, 0, 0
Greece	3	3	-	–	1, 2, 0, 0
Hungary	7	2	5	–	7, 0, 0, 0
Italy	3	–	3	–	3, 0, 0, 0
Poland	5	3	2	–	5, 0, 0, 0
Portugal	4	4	-	–	4, 0, 0, 0
Romania	11	10	1	–	8, 0, 3, 0
Serbia	1	1	–	–	1, 0, 0, 0
Spain	9	9	–	–	4, 3, 0, 2
Switzerland	1	–	1	–	1, 0, 0, 0
The Netherlands	1	–	1	–	1, 0, 0, 0
Total	172	–	-	–	156, 9, 5, 2

^a^It was possible to name more than one country in the questionnaire

### Sequencing

Sequencing was successful for all 172 samples that tested positive in 2018 and 2019. Of these 172 samples, 156 tested positive for *B. canis* (99.80–100.00% identity to GenBank MN134074.1), nine for *B. vogeli* (99.18—100.00% identity to GenBank JX304677.1), five for *B. gibsoni* (99.79–100.00% identity to GenBank MN134517.1) and two for *B. vulpes* (99.04—99.42% identity to GenBank MK585200.1) (Table 4).

## Discussion

The likelihood of a dog testing positive for *Babesia* spp. by PCR increased by about 3% each year, and the number of dogs testing positive varied between the individual years. The following factors likely influenced *Babesia* spp. infections in dogs in the present study: the geographical distribution of *D. reticulatus* ticks in Germany, which is expanding [14, 37–39]; changing climatic conditions in Europe; country of origin; importation into Germany and/or travel from Germany to other countries in Europe, both of which are increasing.

**Fig. 2:**
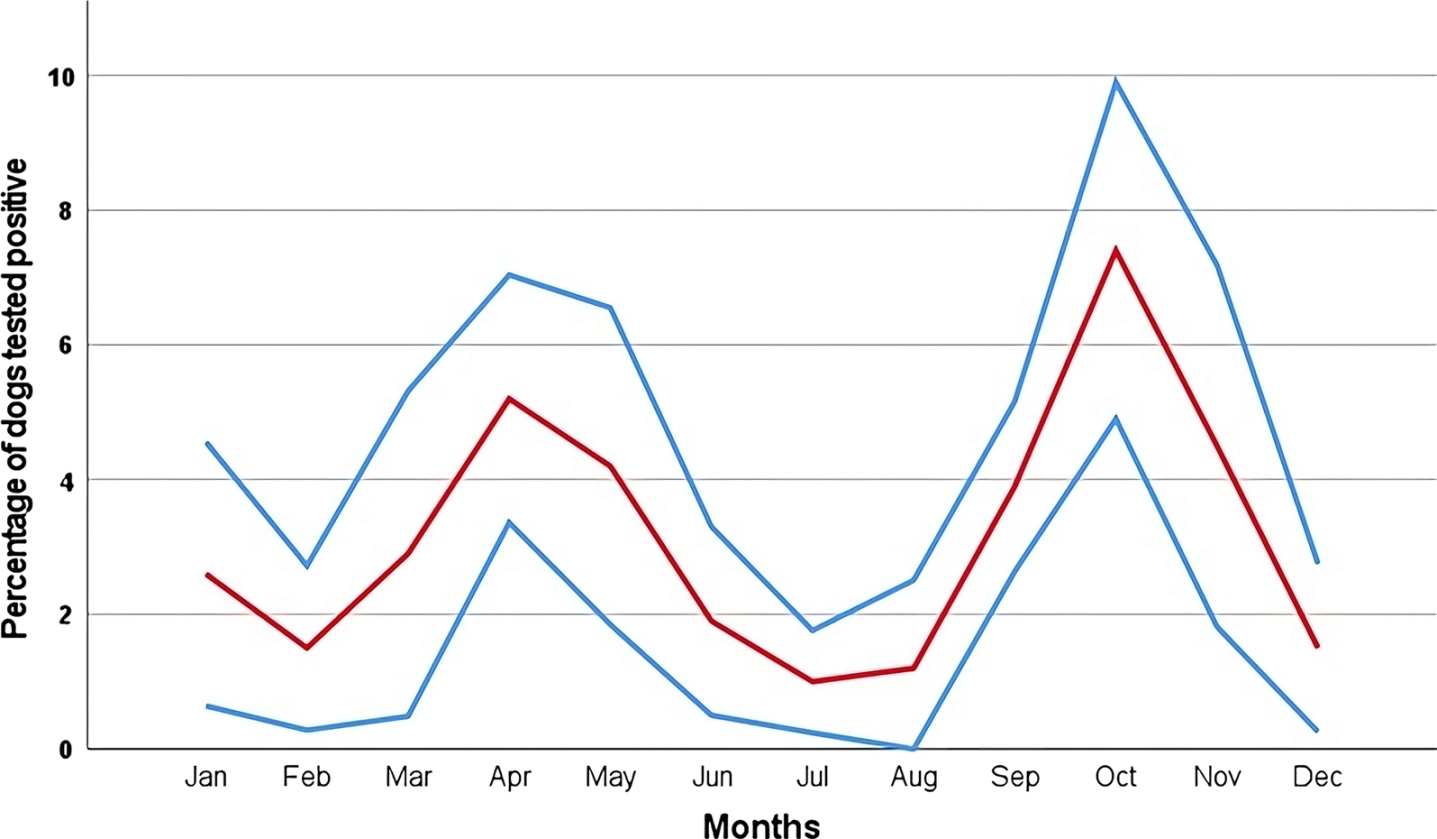
Monthly distribution of percentages of dogs testing positive for *Babesia* spp. by PCR from 2007 to 2020 [mean (*red line*), SD (blue lines)]

The range of *D. reticulatus* is significantly expanding, especially in northern Germany [38]. Several studies [38–40] reported highest numbers of *D. reticulatus* ticks collected from dogs in February and March and in September and October; these data fit well with the results of our study, with peaks in positive PCR tests for *Babesia* spp. in spring and autumn (Fig. 2). A strong association between the occurrence of ticks and canine babesiosis, as well as between seasonal patterns of *D. reticulatus*’ occurrence and outbreaks of canine babesiosis, was demonstrated for Poland [41]. However, the fact that the time of PCR testing in our study may not correspond to the time of infection, and therefore with the time of contact of the dog with the tick, needs to be taken into consideration. However, a positive PCR result for *Babesia* spp. is highly indicative of an acute infection, and the seasonal distribution of the positive test results fits well with the seasonal activity of *D. reticulatus* ticks. Information on the treatment of the dogs prior to sampling, and/or vaccinations, which may have influenced the results of our study, was not available.

A surprisingly high number of infections were detected in dogs that had no stays abroad. The number of autochthonous infections was also high relative to the number of infections in dogs for which stays abroad outside of Germany had been reported. In previous studies, individual case reports of autochthonous *B. canis* infections were mainly reported for southern federal states of Germany, e.g. Baden-Württemberg, Rhineland-Palatinate, Saarland, and Bavaria [8–13, 19]. An outbreak of autochthonous *B. canis* infections in the federal states of Berlin and Brandenburg in northeastern Germany, with the majority of clinical cases presenting between September and November, was recently reported [42]. These results fit well with our data regarding the seasonality of positive *Babesia* spp. test results for dogs living in Germany. Our findings also underline the fact that *Babesia* spp. infections in dogs should be considered a possibility all year-round in Germany, notwithstanding the fact that the highest odds in the present study were for spring and autumn.

**Fig. 3:**
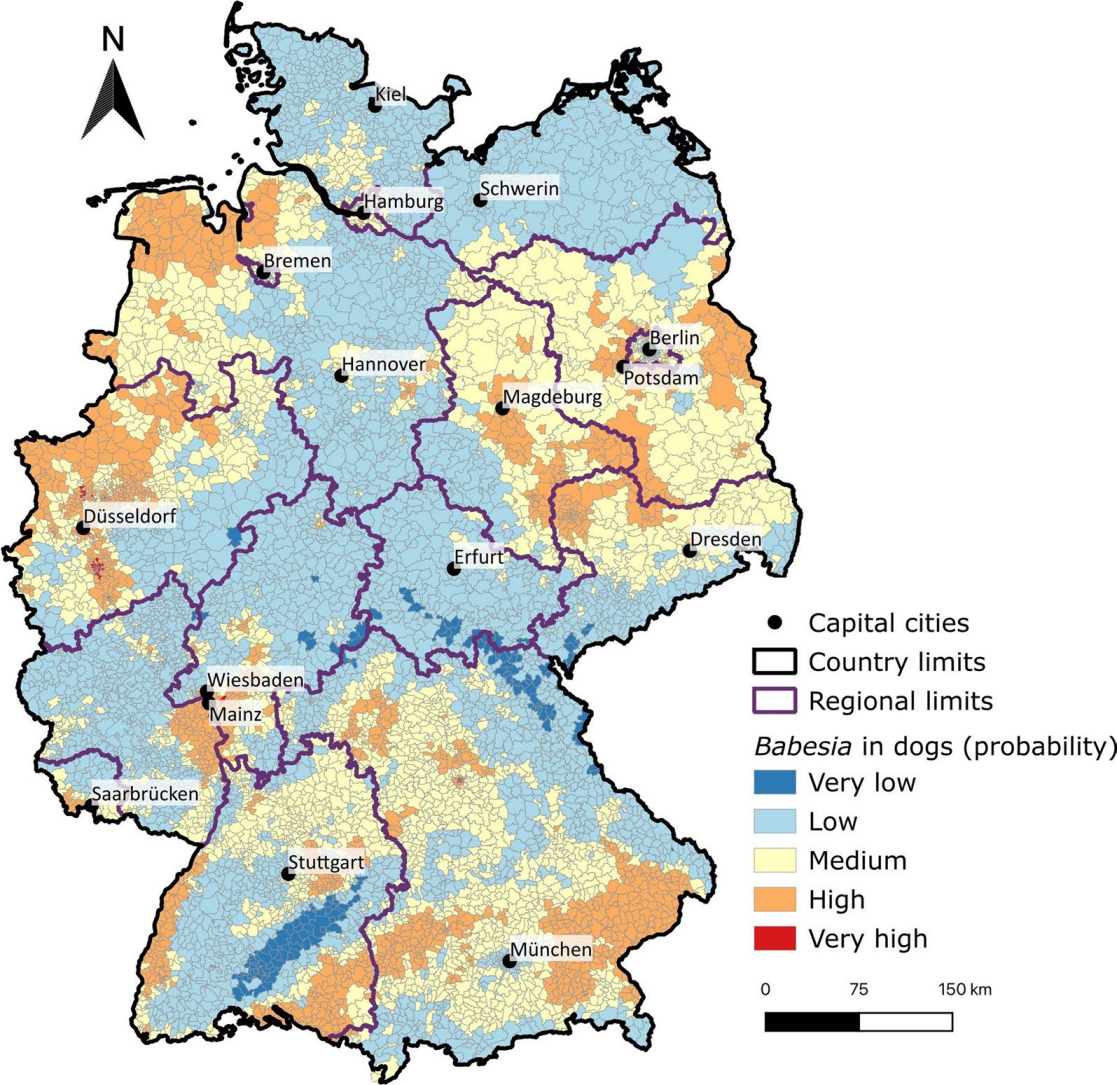
Spatial distribution modelling based on *Babesia* spp.-positive PCR results aggregated according to German five-digit postcodes and classified into five classes

The federal states of Berlin and Brandenburg are known to be areas with a high abundance of *D. reticulatus* in vegetation [40]. In addition, *D. reticulatus* ticks were found on dogs almost as frequently as *I. ricinus* ticks were in the states of Berlin and Brandenburg in another study [43], and at even higher numbers compared to other tick species in other results from northeastern Germany [39]. In our distribution learning model, the likelihood of a positive *Babesia* spp. test result was classified as moderate for northeastern Germany, which is in accordance with the results of a recent tick collection study [39], in which the majority of collected ticks were *D. reticulatus*. However, medium and high likelihoods of dogs testing positive for *Babesia* spp. were predicted for northwestern and southern federal states of Germany (Fig. 3). With respect to these findings, it should be noted that our model relies predominantly on data for dogs with a history of stays abroad, and that it is possible that people living in those areas of Germany travel/vacation more often with their dogs in areas that are endemic for *B. canis*, or are more likely to adopt a dog from an endemic country. However, the overlap between the moderate likelihood of *Babesia* spp.-positive results and longstanding presence *D. reticulatus* in northeastern Germany are also noteworthy findings that potentially support the endemicity of *B. canis* in this region. *Babesia canis* has been occasionally detected in *D. reticulatus* ticks in Germany in southern federal states, i.e. Bavaria [1/301 ticks testing positive (0.3%)] [17], Baden-Wuerttemberg [2/3411 testing positive (0.06%)] [18], and Saarland (10/397 testing positive, 2.5%) [19], but not in northern federal states, e.g. Berlin and Brandenburg [20]. An increase in the distribution of *D. reticulatus* has also been reported in regions in central Europe, e.g. central and eastern Poland, with a predominance of *D. reticulatus* compared *I. ricinus* ticks [44].

There was no statistically significant difference between the *Babesia* spp.-positive test results (*P* = 0.088) of dogs that had stays abroad compared to those that had not. However, this means that there was still a 91.2% chance that there would be a difference between dogs that had versus those that had no stays abroad from Germany. Additionally, only a limited number of dogs with a full medical history were included in our study, as questionnaires were only completed for a limited number of the tested dogs. In 6.9% of the dogs that had never left Germany (69 of 905 dogs) according to the information provided by the treating veterinarian, *Babesia* spp. were identified by PCR, with *B. canis* being the predominant species identified by sequencing. Species differentiation was only performed for left-over samples which dated from 2018 onwards. In general, the detection of *B. canis* in central and eastern European countries is related to the distribution of the vector *D. reticulatus* [2]. This fits with the results of the present study, in which *B. canis* infections were detected in dogs that had travelled from Germany to countries in which* D. reticulatus* is present, including Hungary, Poland, France, Denmark, Switzerland, and the Netherlands.

In the Mediterranean area, *B. vogeli* is another major *Babesia* species that has been identified in dogs [2]. This pathogen is transmitted by *R. sanguineus* ticks, which are thus far considered non-endemic in central, eastern, and northern Europe. This is supported by the fact that infections with* B. vogeli* were only found in dogs that had travelled to the southern European countries of Spain and Greece. *Babesia vulpes* infections were exclusively found in dogs that had travelled to Spain, a country in which a high prevalence of *B. vulpes* has been detected in wildlife, especially in foxes, which were found to have a prevalence as high as 64% [45, 46]. However, the infection of dogs with *B. vulpes* is considered rare. *Babesia gibsoni* infections were detected in three dogs that had stays abroad in Romania. No further information was available regarding potential routes of transmission in these dogs, e.g. vertical transmission [47] or direct contact with other dogs through fighting and bite wounds, or saliva and/or blood ingestion [48–50]. Based on epidemiological data, *B. gibsoni* is thought to be transmitted vectorially, but as this has not been demonstrated naturally in dogs in Europe [2], we think that vectorial transmission in these dogs is unlikely. The highest numbers of dogs that has stays abroad from Germany and tested positive for *Babesia* spp. by PCR had been to Poland (17.9%) and Hungary (13.8%). These results agree well with the reported expansion of the distributions of canine babesiosis and *D. reticulatus* ticks in Poland [3, 44, 53] and the classification of Hungary as a high-risk area [2, 51, 52].

To the best of our knowledge, this is the first study to demonstrate the statistically significant impact of tick attachment (*P* < 0.001) and ectoparasite prophylaxis (*P* = 0.006) on *Babesia* spp.-positive PCR results for dogs that reside in Germany. The importance of ectoparasite prophylaxis for dogs is underlined by the fact that dogs with reported tick attachment had a 7 times higher odds (OR = 7.634), and dogs without ectoparasite prophylaxis 3 times higher odds (OR = 3.020), of testing positive for *Babesia* spp. by PCR. In a previous study [54] on dogs in the states of Berlin and Brandenburg, 92% of the owners reported tick attachment in their dogs, but licensed active ingredients against ticks had only been used in 53% of the dogs. This fits well with the results of our study, in which ectoparasite prophylaxis was reported for 50.8% of dogs with known anamnesis. As *Babesia* spp. infections were documented in each month in our study, year-round ectoparasite prophylaxis is highly recommended.

Male dogs had 46% higher odds (OR = 1.463) of testing positive for *Babesia* spp. by PCR compared to females (Table 2). One possible reason for this is a different level exposure of male and female dogs to vector ticks. Differences between males and females with respect to prevalence, infection intensity, and clinical outcome are frequently observed for parasitic diseases [55]. These differences can be attributed to physiological effects, i.e. the immunosuppressive effects of sex hormones, in particular testosterone, or to behavioural effects, e.g. higher exposure of one sex due to a different habitat preference. For dogs in the present study population, it is impossible to discriminate between these factors. For instance, we do not know how many or which of the dogs were neutered, a procedure that reverses the immunosuppressive effects of testosterone [55]. Thus, it remains unclear why male dogs had a higher predisposition for *Babesia* spp. infections, and further studies are needed to elucidate this.

There was a statistically significant impact of age (*P* < 0.001) on *Babesia* spp.-positive PCR results between dogs under 7 years of age compared to those that were older. Infections with *B. canis*, *B. vogeli*, or *B. rossi* were more frequently seen in young dogs that presented with babesiosis in several studies [5, 56, 57]. To the best of our knowledge, the reasons for the effect of age on infections with *Babesia* spp. have yet to be identified. However, the fact that younger dogs are often more physically active that older dogs may lead to their more frequent contact with ticks and therefore a possibly higher risk of infection.

The limitations of this study are mainly its retrospective design, with missing data regarding the medical records of the dogs, and lack of information regarding why the dogs were tested by PCR for *Babesia* spp., both of which may have had an impact on the percentages of dogs testing positive. Additionally, no information was available for inclusion in the data analysis about the ingredients and duration of ectoparasite prophylaxis, when given, or clinical signs, or the results of blood smear analysis.

## Conclusions

In Germany, possible canine infections with *B. canis* should be especially considered in spring and autumn, as these are the seasons in which the vector *D. reticulatus* is most active there. Travelling with their owners and the importation of dogs are often considered important factors with respect to canine *Babesia* spp. infections in Germany. However, autochthonous infections with *Babesia* spp. also occur in a considerable number of dogs in Germany. Thus, year-round ectoparasite prophylaxis for dogs in Germany in addition to screening for vector-borne infectious pathogens, e.g. *Babesia* spp., in dogs imported into the country are highly recommended. The fact that no ectoparasite prophylaxis was reported for almost half of the dogs in our study indicates a lack of information provided by owners on this and also suggests that some dogs in Germany may be at higher risk of infection with vector-borne pathogens.

### Supplementary Information

**Additional file 1. **Dogs tested by *Babesia* spp. PCR with known countries of stays abroad from Germany according to the questionnaires (*n* positive/*N* total (%)).

## Data Availability

All of the data generated or analyzed during this study are included in this published article.
