# Supplementary material for: Molecular detection of Babesia spp. in dogs in Germany (2007–2020) and identification of potential risk factors for infection
Source: Parasit Vectors. 2023 Nov 2;16:396. doi: 10.1186/s13071-023-06005-7 (PMC10621212; doi:10.1186/s13071-023-06005-7)
Supplement: Supplementary file 1 — Additional file 1. Dogs tested by Babesia spp. PCR with known countries of stays abroad from Germany according to the questionnaires (n positive/N total (%)). [file 13071_2023_6005_MOESM1_ESM.docx]

**Additional file 1:** Named countries in dogs with known anamnesis of stays abroad from Germany (n positive/N total (%))

| **Country** | **Total** | **Import** | **Travel^A^** | **Import and travel^A^** |
| --- | --- | --- | --- | --- |
| Albania | 0/1 (0) | -/- | 0/1 (0) | -/- |
| Austria | 2/51 (3.9) | 0/1 (0) | 2/47 (4.3) | 0/3 (0) |
| Belgium | 0/3 (0) | 0/1 (0) | 0/2 (0) | 0/0 (0) |
| Bosnia and Hercegovina | 2/9 (22.2) | 0/6 (0) | 2/2 (100) | 0/1 (0) |
| Bulgaria | 0/17 (0) | 0/15 (0) | 0/2 (0) | -/- |
| Croatia | 6/46 (13) | 3/15 (20) | 3/29 (10.3) | 0/2 (0) |
| Cyprus | 0/7 (0) | 0/7 (0) | -/- | -/- |
| Czech Republic | 1/18 (5.6) | 0/6 (0) | 0/10 (0) | 1/2 (50) |
| England | 1/11 (9.1) | 0/2 (0) | 1/9 (11.1) | -/- |
| France | 7/85 (8.2) | 2/11 (18.2) | 5/63 (7.9) | 0/11 (0) |
| Greece | 3/59 (5.1) | 3/46 (6.5) | 0/9 (0) | 0/4 (0) |
| Hungary | 11/80 (13.8) | 6/62 (9.7) | 5/14 (35.7) | 0/4 (0) |
| Ireland | 0/2 (0) | 0/1 (0) | 0/1 (0) | -/- |
| Italy | 6/107 (5.6) | 0/22 (0) | 6/80 (7.5) | 0/5 (0) |
| Lithuania | 0/1 (0) | 0/1 (0) | -/- | -/- |
| Malta | 0/5 (0) | 0/5 (0) | -/- | -/- |
| Netherlands | 1/11 (9.1) | 0/2 (0) | 1/9 (11.1) | -/- |
| Poland | 12/67 (17.9) | 6/31 (19.4) | 4/31 (12.9) | 2/5 (40.0) |
| Portugal | 5/23 (21.7) | 4/17 (23.5) | 0/5 (0) | 1/1 (100.0) |
| Romania | 18/135 (13.3) | 16/132 (12.1) | 2/2 (100.0) | 0/1 (0) |
| Serbia | 2/6 (33.3) | 2/4 (50.0) | 0/2 (0) | -/- |
| Scandinavia | 3/58 (5.2) | 0/3 (0) | 3/53 (5.7) | 0/2 (0) |
| Slovakia | 0/3 (0) | 0/2 (0) | 0/1 (0) | -/- |
| Slovenia | 0/6 (0) | 0/3 (0) | 0/2 (0) | 0/1 (0) |
| Spain | 6/168 (3.6) | 6/129 (4.7) | 0/33 (0) | 0/6 (0) |
| Switzerland | 1/12 (8.3) | 0/1 (0) | 1/11 (9.1) | -/- |
|  |  |  |  |  |
| Belarus | 0/3 (0) | 0/3 (0) | -/- | -/- |
| Brasilia | 0/2 (0) | -/- | -/- | 0/2 (0) |
| Chile | 0/2 (0) | 0/1 (0) | -/- | 0/1 (0) |
| Costa Rica | 0/1 (0) | 0/1 (0) | -/- | -/- |
| Dubai | 0/1 (0) | 0/1 (0) | -/- | -/- |
| Israel | 0/3 (0) | 0/1 (0) | -/- | 0/2 (0) |
| Japan | 0/1 (0) | 0/1 (0) | -/- | -/- |
| Korea | 0/1 (0) | 0/1 (0) | -/- | -/- |
| Mauritius | 0/2 (0) | 0/2 (0) | -/- | -/- |
| Philippines | 0/1 (0) | -/- | 0/1 (0) | -/- |
| Russia | 2/9 (22.2) | 1/6 (16.7) | 1/2 (50.0) | 0/1 (0) |
| South Africa | 0/1 (0) | -/- | 0/1 (0) | -/- |
| Sri Lanka | 3/5 (60.0) | 3/5 (60.0) | -/- | -/- |
| Syria | 0/1 (0) | 0/1 (0) | -/- | -/- |
| Thailand | 0/5 (0) | 0/5 (0) | -/- | -/- |
| Turkey | 0/12 (0) | 0/8 (0) | 0/4 (0) | -/- |
| Ukraine | 1/12 (8.3) | 0/6 (0) | 1/5 (20.0) | 0/1 (0) |
| USA | 0/9 (0) | 0/5 (0) | 0/2 (0) | 0/2 (0) |

^A^It was possible to name more than one country in the questionnaire
